# Apoptosis-driven chromosomal rearrangements in cancer: CAD cleavage, nuclear architecture, and microhomology-mediated end joining

**DOI:** 10.1038/s41389-026-00645-x

**Published:** 2026-07-24

**Authors:** Sang-Nee Tan

**Affiliations:** Faculty of Medicine and Health Sciences, Department of Paraclinical Sciences, Universiti Malaysia Sarawak, Kota Samarahan, Malaysia

**Keywords:** Cancer genetics, Apoptosis, Genomic instability, Non-homologous-end joining, Nuclear organization

## Abstract

Chromosomal rearrangements are hallmarks of many cancers, yet the mechanisms governing breakpoint selection remain incompletely understood. Emerging evidence suggests that sublethal apoptotic signaling may contribute to structural genome remodeling. Oxidative stress and inflammatory stimuli trigger caspase activation and the release of caspase-activated DNase (CAD), which preferentially cleaves DNA at matrix association regions/scaffold attachment regions (MAR/SARs) that anchor chromatin loops to the nuclear scaffold. When apoptotic execution is incomplete and cells undergo recovery, a process termed anastasis, CAD-induced double-strand breaks (DSBs) can be repaired through error-prone end-joining pathways, including classical non-homologous end joining (cNHEJ) and microhomology-mediated end joining (MMEJ), producing rearrangements enriched at MAR/SAR-associated genomic regions. Drawing on mechanistic studies in leukemia and experimental evidence in nasopharyngeal carcinoma (NPC), this review outlines a framework linking apoptotic DNA fragmentation, nuclear architecture, and error-prone repair to recurrent structural variations, and discusses how shared inflammation- and virus-associated oxidative stress may promote analogous mechanisms of breakpoint formation in other malignancies. The convergence of findings across hematologic and epithelial malignancies strengthens the biological plausibility and potential generalizability of this model across inflammation- and stress-associated cancers. This perspective suggests that at least a subset of cancer-associated chromosomal rearrangements may arise not purely as stochastic by-products of genomic instability, but as structured outcomes of dysregulated cell-death programs acting on vulnerable chromatin domains. Beyond its mechanistic implications, this framework may inform biomarker discovery through apoptosis-associated breakpoint features and highlight potential therapeutic vulnerabilities in apoptosis-surviving cells, including dependence on polymerase theta-mediated repair or regulation of apoptotic execution.

## Introduction

Apoptosis is classically regarded as a tumor-suppressive program that eliminates damaged cells [1]. However, accumulating evidence indicates that incomplete apoptotic execution can paradoxically generate clonally heritable genomic alterations [2, 3]. Chromosomal rearrangements are hallmark features of many cancers, exemplified by recurrent *MLL* translocations in hematologic malignancies [2, 4–6] and by regionally clustered copy number gains and losses in epithelial tumors such as nasopharyngeal carcinoma (NPC) [7–10]. However, the mechanisms governing breakpoint selection remain incompletely understood. Recurrent structural variants often cluster within defined genomic regions, as illustrated by breakpoint cluster regions (BCRs) in *MLL* and matrix association regions/scaffold attachment regions (MAR/SAR)-enriched loci in NPC [5, 6, 11–13], suggesting that non-random chromatin contexts may shape breakpoint formation during tumor evolution.

Conventional models attribute mutagenesis primarily to reactive oxygen species (ROS)-induced DNA lesions that are repaired by base excision repair (BER) or non-homologous end joining (NHEJ). While these mechanisms explain random mutations, they do not readily account for the recurrent localization of breakpoints at specific genomic hotspots or the non-random emergence of structural alterations [14, 15]. These observations raise the possibility that defined chromatin domains, rather than stochastic DNA damage alone, contribute to breakpoint selection.

Mechanistic studies in leukemia first provided direct evidence linking apoptotic DNA fragmentation to oncogenic chromosomal rearrangements. The apoptotic nuclease caspase-activated DNase (CAD/DFF40) generates double-strand breaks (DSBs) within the *MLL* breakpoint cluster region (BCR), frequently at MAR/SARs and DNase I hypersensitive sites [5, 6]. Apoptotic stimuli, including death receptor ligation, irradiation, and chemotherapeutic exposure, induce CAD-dependent DSBs that can be misrepaired by NHEJ to form leukemogenic translocations [2, 4, 16]. These findings established a mechanistic basis in which apoptotic nuclease activation, higher-order chromatin organization, and error-prone end joining cooperate to generate structural rearrangements.

Similar apoptotic breakage patterns have been observed in epithelial solid tumors, particularly in NPC, a malignancy strongly associated with Epstein-Barr virus (EBV) infection and chronic inflammation [12, 13, 17–20]. In this context, oxidative stress-induced apoptosis produces site-specific DNA cleavage and microhomology-associated rearrangements [12, 13, 17–20]. NPC exhibits recurrent chromosomal losses and gains that cluster within defined genomic regions, including deletions at 3p, 9p, and 11q and amplifications at 1q, 3q, and 8q [7–10]. The non-random distribution of these alterations raises the possibility that chromatin architecture and apoptotic DNA fragmentation contribute to structural variant formation in solid tumors.

NPC etiology is multifactorial, involving EBV infection [21, 22], genetic susceptibility [10], and environmental exposures such as nitrosamine-rich diets and wood dust [23]. In addition, gastroesophageal reflux may expose the nasopharyngeal epithelium to bile acids, contributing to chronic rhinosinusitis and increased NPC risk [18, 24]. A common biological theme across these factors is chronic inflammation and oxidative stress in the nasopharynx [20], conditions known to promote DNA damage [25, 26]. Similar inflammatory or virus-associated contexts are present in other malignancies, including Barrett’s esophagus, colitis-associated colorectal cancer, hepatocellular carcinoma (HCC), gastric cancer, bladder cancer, and EBV-associated lymphomas [27–32]. These shared stress environments raise the possibility that sublethal apoptotic signaling and incomplete execution may contribute to recurrent breakpoint formation in diverse tumor types, consistent with experimental evidence that transient or reversible apoptotic responses can permit DNA damage persistence and oncogenic transformation [1, 33, 34], and that caspase-dependent activation of CAD contributes to mutagenesis in surviving cells [16].

Apoptosis normally culminates in orderly cellular elimination. However, under certain conditions, cells can survive and recover from the execution phase, a process termed anastasis [1, 33]. Surviving cells can retain DNA breaks and micronuclei, and exhibit chromosomal instability [1, 35, 36], suggesting that incomplete apoptosis itself may contribute to mutagenesis [1, 34]. Experimental studies in NPC cell models demonstrate that oxidative stress-induced apoptosis generates CAD-mediated DSBs preferentially at MAR/SARs, which are subsequently repaired through error-prone end joining, including NHEJ and microhomology-mediated end joining (MMEJ), producing rearrangements that mirror those observed in tumors [12, 13, 18–20].

The principal nuclease responsible for apoptotic DNA fragmentation is CAD (DFF40), which is normally inhibited by ICAD (DFF45) [37, 38]. Upon apoptotic stimulation, executioner caspases, particularly caspase-3, cleave ICAD, thereby releasing active CAD to mediate chromatin DNA cleavage [38, 39].

In addition to generating widespread DNA fragmentation, apoptotic DNA cleavage exhibits non-random and context-dependent features that suggest preferential targeting of specific chromatin domains. Importantly, CAD-mediated cleavage does not occur uniformly across the genome but is influenced by higher-order chromatin organization and local DNA structural features. Early biochemical and nuclear matrix studies demonstrated that apoptotic DNA fragmentation proceeds through an initial excision of large chromatin loops (approximately 50-300 kb) at MAR/SARs, followed by further processing into nucleosomal fragments [40]. MAR/SAR elements are typically AT-rich, structurally flexible sequences that anchor chromatin loops to the nuclear scaffold and are often associated with increased DNA accessibility, including DNase I hypersensitive sites and topoisomerase II cleavage regions [41–44].

Consistent with these structural properties, multiple studies have shown that apoptotic DNA cleavage is enriched at genomic loci characterized by MAR/SAR features and open chromatin configurations. In leukemia models, cleavage within the *MLL* breakpoint cluster region (BCR) occurs at sites that coincide with scaffold attachment regions (SARs) and DNase I hypersensitive sites [5, 6], while similar enrichment of cleavage at MAR/SAR-associated regions has been observed in NPC models under oxidative stress [12, 13, 19]. These observations suggest that CAD-mediated cleavage is associated with chromatin organization, with loop anchor regions and structurally accessible DNA domains representing preferential sites of apoptotic DNA cleavage.

Together, these findings support a model in which apoptotic DNA cleavage is not purely stochastic but is shaped by nuclear architecture and chromatin context. In this framework, MAR/SAR-associated chromatin domains represent structurally vulnerable regions where CAD-mediated cleavage is more likely to occur, thereby contributing to the non-random distribution of breakpoints observed in cancer-associated chromosomal rearrangements. This framework provides a mechanistic basis for understanding breakpoint clustering in cancer genomes as a consequence of structured DNA damage occurring at defined chromatin domains, complementing models based on stochastic genomic instability.

**Fig. 1:**
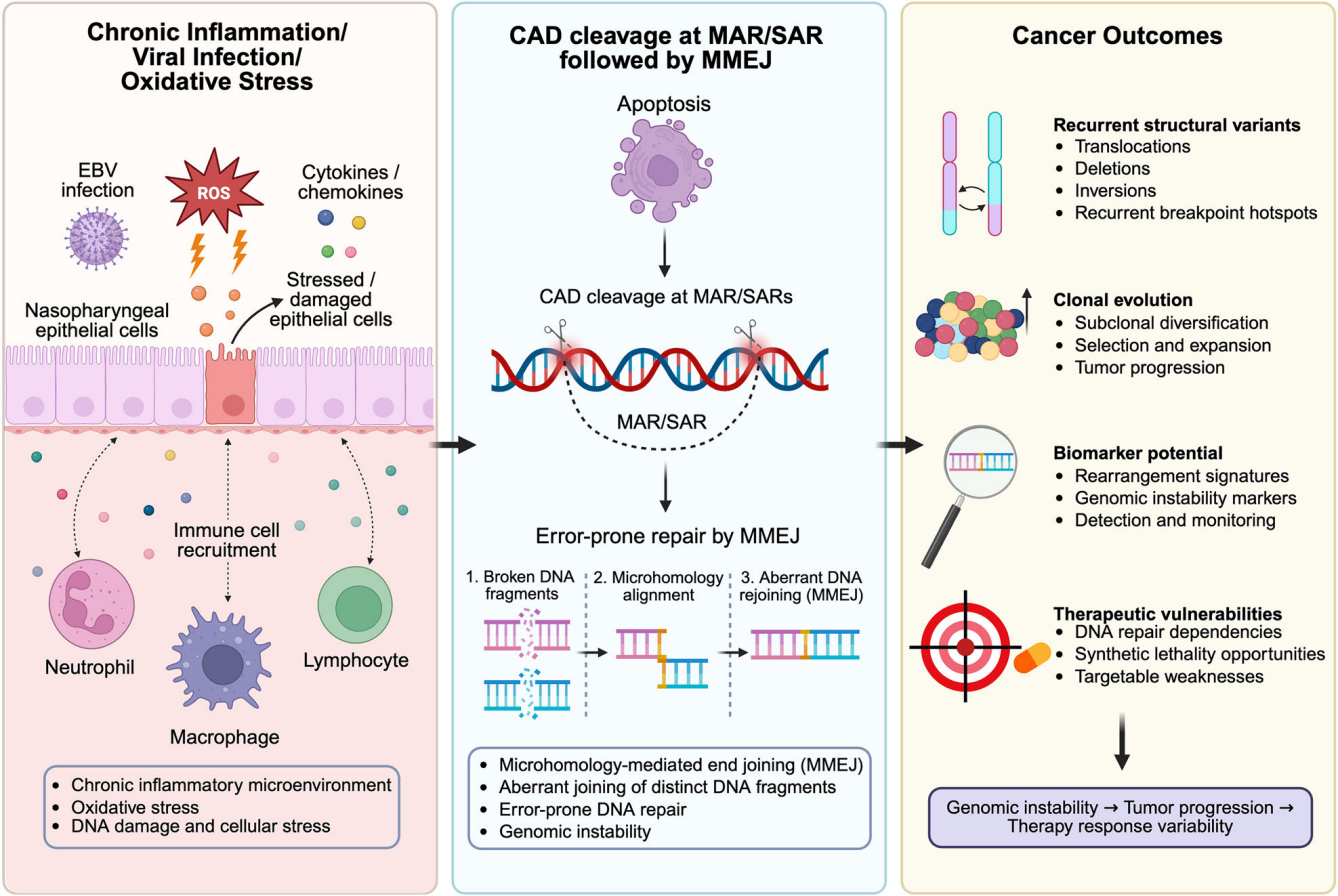
Conceptual overview of apoptosis-induced chromosomal rearrangements in cancer. Chronic inflammation and viral infection promote oxidative stress, inflammatory signaling, and cellular stress in epithelial cells within a chronically inflamed tissue microenvironment, illustrated here using nasopharyngeal epithelial cells as a representative example (left). These stress-associated conditions can trigger apoptosis and activate caspase-dependent apoptotic execution programs. During apoptosis, caspase-dependent cleavage of ICAD releases active CAD, which induces DNA cleavage at chromatin regions associated with MAR/SAR elements. In cells that survive incomplete apoptosis, including through apoptotic recovery (anastasis), CAD-induced DNA double-strand breaks (DSBs) may undergo error-prone repair through MMEJ or MMEJ-like repair, involving microhomology alignment and aberrant rejoining between distinct DNA fragments (middle). These processes may contribute to genomic instability and the formation of recurrent structural variants, including translocations, deletions, inversions, and recurrent breakpoint hotspots. Accumulation of these structural variants may promote clonal evolution through subclonal diversification, selection and expansion of rearrangement-bearing clones, and tumor progression. The resulting rearrangement patterns and DNA repair dependencies may also provide opportunities for biomarker discovery and therapeutic targeting in inflammation- and virus-associated cancers characterized by chronic oxidative stress (right). Created using Canva Pro and BioRender. Created in BioRender. TAN, S. N. (2026) https://BioRender.com/dfwpgf2.

This review synthesizes mechanistic evidence from leukemia and NPC to outline a framework linking apoptotic DNA fragmentation, nuclear architecture, and error-prone repair to recurrent structural variations. It further examines how inflammation- and virus-associated oxidative stress may promote analogous mechanisms across cancer types, and discusses implications for predicting structural variant hotspots and therapeutic strategies targeting vulnerabilities created by apoptosis-associated misrepair. By reframing certain chromosomal rearrangements as structured outcomes of dysregulated cell-death programs rather than purely stochastic genomic instability, this perspective offers new insight into cancer evolution and opportunities for intervention, as summarized in Fig. 1.

## Apoptotic DNA cleavage and chromosomal rearrangements in leukemia

### Site-specific apoptotic cleavage within the *MLL* breakpoint cluster region

Leukemia studies provided the first mechanistic evidence linking apoptotic DNA fragmentation to oncogenic chromosomal rearrangements [2, 4–6, 34]. Translocations involving the *MLL* gene on chromosome 11q23 are characteristic of therapy-related acute leukemias and cluster within a defined 8.3-kb breakpoint cluster region (BCR) [6, 11]. Early analyses demonstrated that DNA cleavage within the *MLL* BCR occurs during apoptosis and coincides with higher-order chromatin fragmentation [6]. Site-specific cleavage at a locus adjacent to exon 12 within the *MLL* BCR was observed following treatment with topoisomerase II inhibitors as well as non-topoisomerase-targeting chemotherapeutic agents and non-genotoxic apoptotic stimuli, suggesting that this cleavage represents a generalized apoptotic response rather than a drug-specific topoisomerase-mediated event [5, 6].

Importantly, drug-induced DNA cleavage within the *MLL* BCR was shown to be nuclease-mediated and caspase-dependent. Cleavage kinetics paralleled nucleosomal DNA ladder formation, was not protein-linked, and were abolished by caspase inhibition, supporting the involvement of an apoptotic nuclease rather than direct topoisomerase II cleavage [5].

### CAD-dependent double-strand breaks and error-prone end joining

These findings were further substantiated by genetic evidence implicating CAD. In models of etoposide-induced treatment-related acute myelogenous leukemia (t-AML), CAD was required for efficient formation of *MLL* rearrangements. Etoposide stimulated *MLL* fusion product formation and plasmid integration in CAD-complemented cells but not in CAD-deficient cells [4]. Downregulation of ICAD, the inhibitor and folding chaperone of CAD, similarly reduced etoposide-induced integration events [4]. These studies established CAD as a critical mediator of apoptosis-associated DSBs within the *MLL* locus.

Translocation junctions generated under apoptotic conditions frequently contained short regions of microhomology, consistent with NHEJ-mediated repair [2]. DNA-dependent protein kinase catalytic subunit (DNA-PKcs), a core NHEJ component, was detected at sites of apoptotic cleavage within *MLL*, and pharmacologic suppression of DNA-PKcs compromised DNA end joining and altered translocation formation [2]. These data indicate that apoptotic nuclease-induced DSBs can be misrepaired by NHEJ to generate leukemogenic translocations. Subsequent studies demonstrated that executioner caspases and CAD are also required for mutagenesis induced by TNF-related apoptosis-inducing ligand (TRAIL)-mediated death receptor ligation and by certain chemotherapeutic agents, including vincristine and topoisomerase poisons, further supporting a role for sublethal apoptotic signaling in mutation generation [16].

### Chromatin structural features and survival after apoptotic cleavage

Chromatin structural analyses of leukemia-associated breakpoint cluster regions (BCRs), including the *MLL* BCR, provided additional mechanistic insight. The *MLL* BCR and its common partner genes, including *AF9* and *AF4*, contain DNase I hypersensitive sites, topoisomerase II cleavage sites, and scaffold attachment regions (SARs) that coincide with patient breakpoint clusters [43, 44]. In *AF9*, two SARs flank patient BCRs and colocalize with topoisomerase II and DNase I hypersensitive sites [44]. Similar structural features were identified across multiple leukemia-associated breakpoint loci, whereas such elements were absent or markedly reduced in homologous genes not involved in translocations, such as *MLL2*, a homolog to *MLL* that is not associated with chromosome translocations [43]. These findings support a model in which higher-order chromatin organization and matrix-associated domains create structural contexts that predispose specific genomic regions to apoptotic cleavage and nonhomologous recombination. Detailed mapping of patient breakpoints further demonstrated nonrandom distribution within the *MLL* BCR. Breakpoints in de novo leukemia preferentially localized to the centromeric half of the BCR, whereas those in therapy-related AML were enriched within the telomeric half, a difference that reached statistical significance [11]. Importantly, SARs and topoisomerase II consensus sites were unevenly distributed across these subregions, suggesting that local chromatin architecture may influence breakpoint selection and that distinct mechanisms may underlie translocations in de novo versus therapy-related leukemia [11].

Accumulating evidence indicates that apoptotic execution is not invariably terminal. Cells exhibiting caspase activation and CAD-mediated chromatin cleavage may survive under certain conditions, potentially through modulation by inhibitors of apoptotic proteins (IAPs) [34]. Translocations initiated at apoptotic nuclease cleavage sites within *MLL* have been detected in surviving cells, suggesting that recovery from the execution phase of apoptosis can permit persistence of apoptosis-induced DNA damage [34]. Together, these studies support a pathogenic model in which apoptotic nuclease activation, locus-specific chromatin architecture, and error-prone DNA repair cooperate to generate oncogenic rearrangements in leukemia, providing a mechanistic framework for considering apoptosis-associated DNA fragmentation as a structured contributor to chromosomal rearrangements.

## Mechanistic model and experimental evidence in NPC

While leukemia provided the first mechanistic evidence that apoptotic nuclease activation can generate locus-specific chromosomal rearrangements [2, 4], an important question is whether similar principles operate in epithelial solid tumors arising in chronic oxidative or inflammatory stress environments that promote recurrent apoptotic signaling. Unlike therapy-related leukemias, NPC develops in a microenvironment characterized by persistent oxidative stress and virus-associated inflammation [20, 21]. These conditions promote apoptotic signaling and, under sublethal stress, may permit cellular recovery [1, 20], raising the possibility that CAD-mediated cleavage at MAR/SAR-defined chromatin domains may also contribute to structural variation in solid tumors [13, 43].

Nasopharyngeal carcinoma (NPC) is linked to chronic exposures such as EBV infection, dietary N-nitrosamines, occupational inhalants, cigarette smoke, and persistent inflammation [45, 46]. These exposures induce oxidative stress [20], which in turn triggers intrinsic apoptosis in nasopharyngeal epithelial cells, characterized by mitochondrial outer membrane permeabilization, loss of mitochondrial membrane potential (MMP), caspase activation, and phosphatidylserine (PS) externalization [12, 18, 20].

### Apoptotic DNA cleavage and nuclear architecture

Caspase-activated DNase (CAD/DFF40) is a key nuclease in the execution phase of apoptosis and is normally sequestered by its inhibitor ICAD (DFF45). In healthy cells, ICAD acts both as a folding chaperone and as an inhibitor of CAD. Upon caspase-3 activation, ICAD is cleaved, thereby releasing CAD to oligomerize and translocate into the nucleus, where it fragments chromatin DNA into ~180 bp nucleosomal units [37]. This cleavage of ICAD therefore represents a decisive checkpoint ensuring that apoptotic genome fragmentation occurs only after caspase activation.

Live-cell imaging revealed that CAD’s nuclear dynamics differ between normal and apoptotic states. In dividing cells, CAD–GFP is diffusely distributed, but in apoptosis CAD rapidly translocates to the nucleus and becomes immobilized on chromatin, coinciding with MAR/SARs [39].

Classical nuclear matrix studies further showed that apoptotic DNA cleavage occurs in two sequential stages, involving first the excision of large 50–300 kb chromatin loops at scaffold attachment sites and second the digestion of these fragments into nucleosomal ladders by CAD [40]. MAR/SARs are AT-rich sequences that tether chromatin loops to the nuclear matrix [41, 42]. Their unwinding and bendable properties make them highly accessible to nucleases [47]. Importantly, MAR/SAR elements often coincide with fragile sites and patient breakpoint clusters. For example, a high proportion of predicted MAR/SAR motifs in *ABL1* overlaps with reported leukemia breakpoints, with similar enrichment observed at *MLL*, *AF9*, and *AF4* [11, 13, 43, 44]. In NPC models, oxidative stress-induced apoptosis preferentially cleaves MAR/SAR-rich intronic regions of tumor suppressors such as *AF9* and *ABL1* [12, 13].

### Error-prone repair and anastasis

If apoptosis proceeds to completion, these breaks are removed with the dying cell. However, some cells can survive transient apoptotic stimuli through a process termed anastasis [1, 34]. Because homologous recombination (HR) is generally unavailable in the absence of sister chromatids, repair is therefore mediated through error-prone end-joining pathways, including classical non-homologous end joining (c-NHEJ) and microhomology-mediated end joining (MMEJ), which can generate deletions and short microhomologies (typically 2–10 bp) at breakpoint junctions [48–51].

**Fig. 2:**
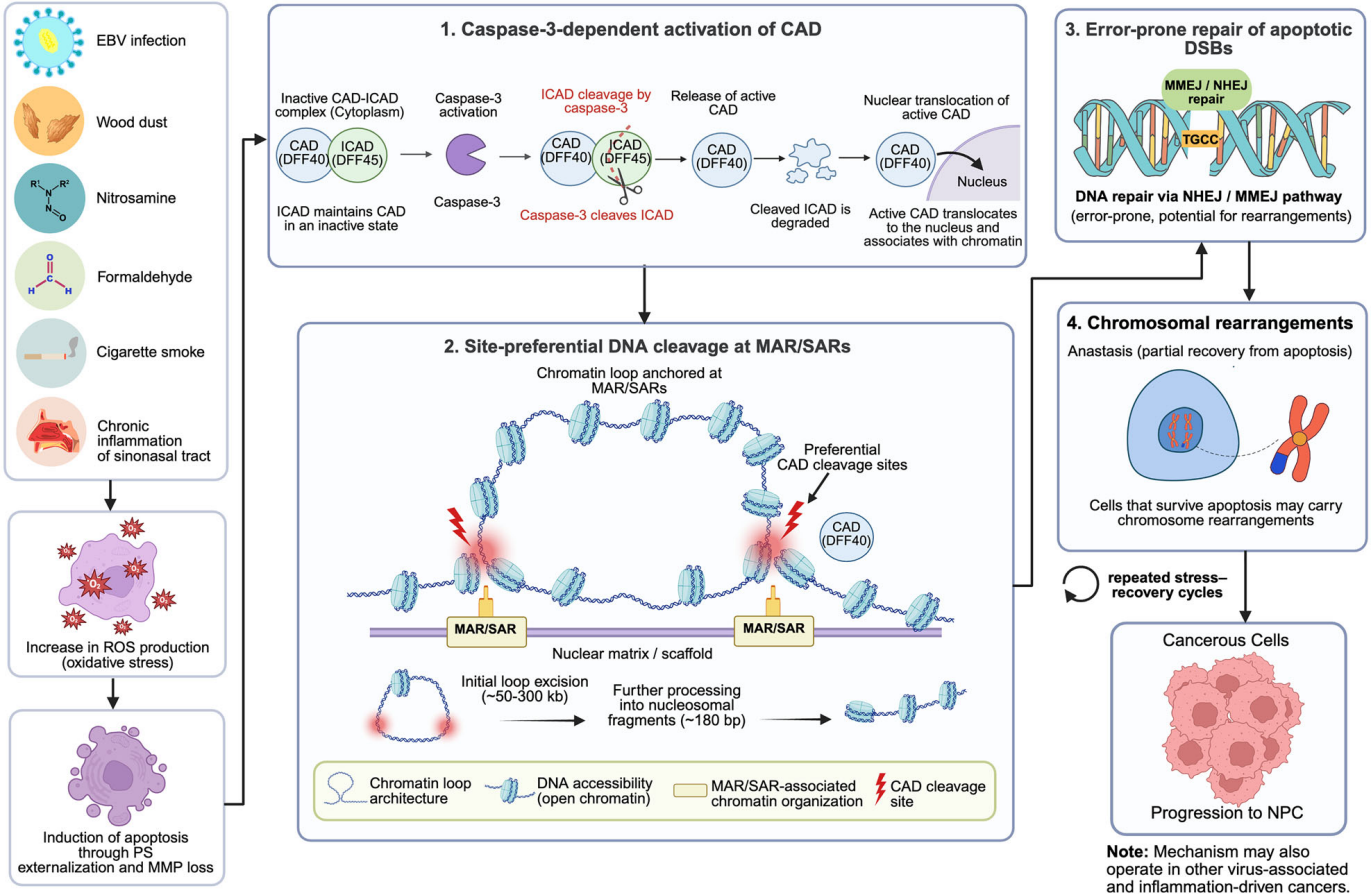
Schematic model of apoptosis-induced chromosomal rearrangements in NPC and related cancers. Apoptotic stimuli, including viral infection (e.g., EBV), environmental carcinogens (nitrosamines, wood dust, formaldehyde), cigarette smoke, and chronic inflammation, induce oxidative stress and increase reactive oxygen species (ROS), thereby triggering apoptosis in nasopharyngeal epithelial cells. Apoptotic signaling, including phosphatidylserine (PS) externalization and mitochondrial membrane potential (MMP) loss, activates caspase-3-dependent apoptotic pathways. (1) Caspase-3–dependent activation of CAD. During apoptosis, caspase-3 cleaves the inhibitor of CAD (ICAD), thereby releasing active CAD (DFF40) from the CAD–ICAD complex. Activated CAD translocates into the nucleus and associates with chromatin. (2) Site-preferential DNA cleavage at MAR/SARs. CAD preferentially cleaves chromosomal DNA at MAR/SARs, which are associated with chromatin loop anchoring, increased DNA accessibility, and AT-rich sequences. This process generates DSBs, initially producing large chromatin loop fragments (~50–300 kb), followed by further processing into nucleosomal DNA fragments (~180 bp). (3) Error-prone repair of apoptotic DSBs. In cells that survive incomplete apoptosis, including through apoptotic recovery (anastasis), CAD-induced DSBs may be repaired through error-prone end-joining pathways, including NHEJ and MMEJ/MMEJ-like repair, with increasing evidence supporting the involvement of microhomology-associated end joining in apoptosis-associated chromosomal rearrangements. Short regions of microhomology (e.g., TGCC) may facilitate alignment and aberrant rejoining of distinct DNA fragments during end-joining repair. (4) Chromosomal rearrangements. Misrepair of apoptotic DSBs can generate chromosomal translocations, deletions, inversions, and other structural variants, thereby promoting genomic instability. Surviving cells may progressively accumulate structural variants during repeated cycles of apoptotic stress and partial recovery, potentially contributing to malignant progression and progression to NPC. Although illustrated here in NPC, this apoptosis–MAR/SAR–MMEJ model may also broadly apply to other virus-associated and inflammation-driven cancers. Created using Canva Pro and BioRender. Created in BioRender. TAN, S. N. (2026) https://BioRender.com/v2ci47k.

Consistent with the mechanistic precedent established in leukemia, classical cytogenetic studies showed that apoptosis in lymphoblasts induces *MLL* translocations containing short regions of microhomology, while inhibition of DNA-PKcs reduces these rearrangements [2]. Deep sequencing studies have further shown that, particularly in HR-deficient contexts, apoptosis-associated DNA breaks frequently exhibit repair signatures consistent with alternative end-joining mechanisms, including MMEJ [52–54]. In NPC cells, rearrangements similarly bear NHEJ/MMEJ-associated signatures and localize to recurrent breakpoint regions enriched for MAR/SAR elements [12]. Collectively, these findings support a broader model in which sublethal apoptosis, CAD-mediated cleavage at MAR/SAR-associated chromatin domains, and error-prone repair contribute to recurrent chromosomal rearrangements in NPC (Fig. 2).

Mechanistically, apoptotic DNA fragmentation generates heterogeneous DNA ends that may undergo limited end resection or end processing, thereby exposing short regions of microhomology. These microhomologous sequences can facilitate alignment of DNA ends prior to ligation, often resulting in deletions, insertions, or chromosomal rearrangements. Such features are characteristic of MMEJ-like repair and are commonly observed at breakpoint junctions in cancer genomes [5, 6, 55].

**Fig. 3:**
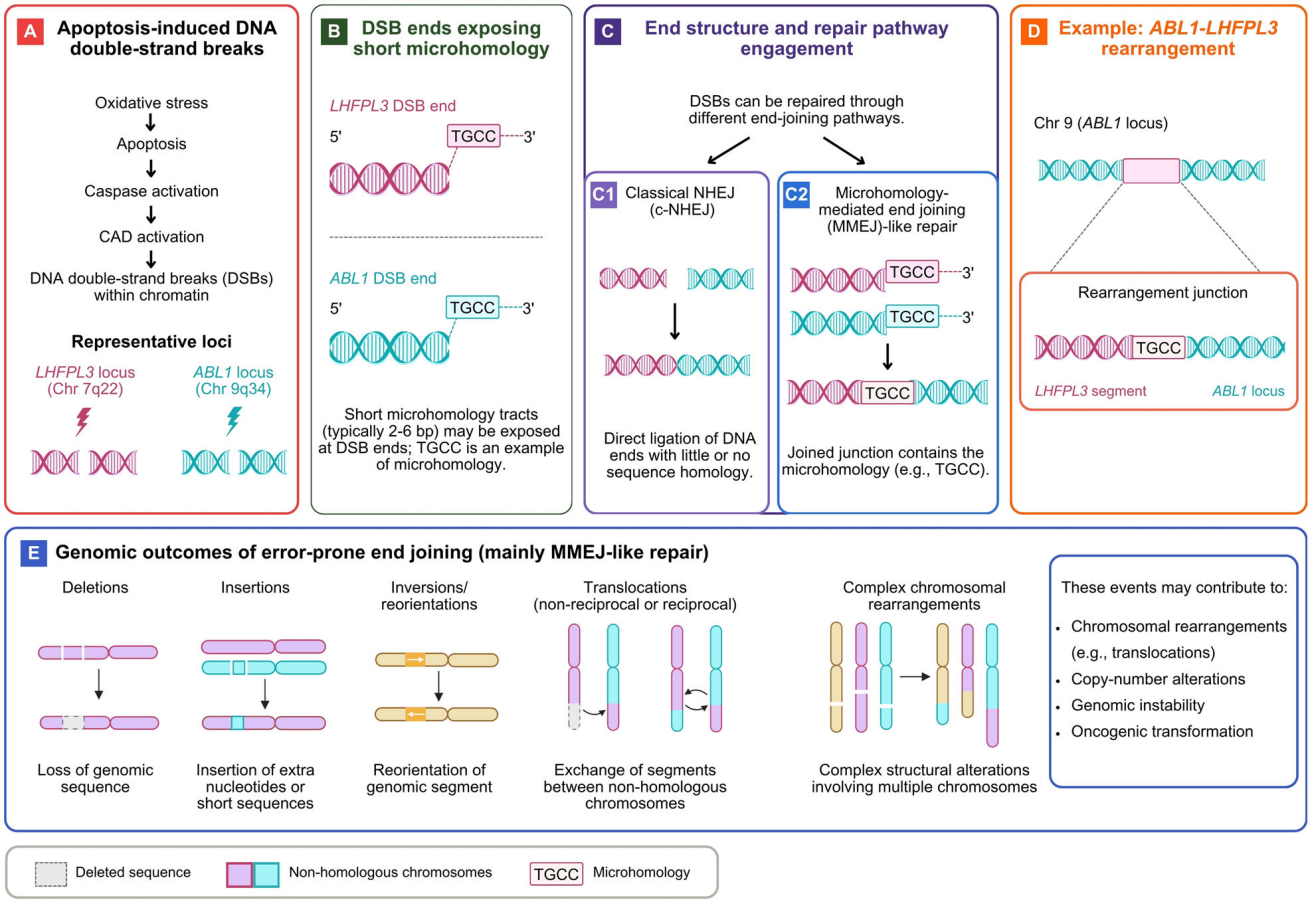
Mechanisms of end-joining repair of apoptosis-induced DNA double-strand breaks. A schematic model illustrating how apoptosis-induced DNA double-strand breaks (DSBs) are processed and repaired through distinct end-joining pathways, leading to diverse genomic outcomes. **A** During apoptosis, caspase-dependent activation of caspase-activated DNase (CAD) induces DSBs within chromatin regions, including representative loci such as *ABL1* and *LHFPL3*. **B** These breaks may generate DNA ends containing short regions of microhomology. TGCC is shown as an example of short microhomology identified at an apoptosis-associated breakpoint junction. **C** DNA end structure may influence repair pathway engagement. Classical non-homologous end joining (c-NHEJ) mediates direct ligation with minimal or no sequence homology, whereas microhomology-mediated end joining-like repair (MMEJ-like repair) utilizes short microhomologous sequences exposed at DNA ends during end-joining repair to facilitate alignment and rejoining of DNA ends. **D** An example is shown for *ABL1*–*LHFPL3*, representing a microhomology-associated chromosomal rearrangement supported by experimental observations of short microhomology at breakpoint junctions in nasopharyngeal epithelial and NPC cell models [12]. **E** Error-prone end joining, mainly involving MMEJ-like repair, can generate deletions, insertions, inversions/reorientations, translocations, and complex chromosomal rearrangements. Representative chromosomal rearrangements are illustrated schematically. Created using Canva Pro and BioRender. Created in BioRender. TAN, S. N. (2026) https://BioRender.com/6023xzp.

Importantly, experimental evidence from nasopharyngeal epithelial and NPC cell models provides direct support for this mechanism. Oxidative stress-induced apoptosis has been shown to induce chromosomal breakage within the *ABL1* gene, followed by non-reciprocal rearrangement events involving sequences from the *LHFPL3* locus. Notably, short regions of microhomology (e.g., TGCC) were identified at breakpoint junctions (Fig. 3), supporting the involvement of an MMEJ-like repair process rather than precise DNA repair [12, 13, 19, 20].

Together, these observations further support a mechanistic framework in which sublethal apoptosis, CAD-mediated cleavage at MAR/SAR-associated chromatin domains, and error-prone end joining cooperate to generate recurrent chromosomal rearrangements. Depending on the extent of end processing and local chromatin context, apoptosis-induced DSBs may be repaired through c-NHEJ or MMEJ-like pathways, leading to structurally diverse outcomes, including deletions, insertions, translocations, and other chromosomal rearrangements. These repair mechanisms and the resulting chromosomal rearrangements are schematically illustrated in Fig. 3.

### NPC-specific experimental evidence

#### CAD-dependent DNA breaks

Oxidative stress induced by hydrogen peroxide (H₂O₂) triggers caspase-3 activation and DNA fragmentation in NPC (SUNE1, HK1), and normal nasopharyngeal epithelial (NP69) cells, with CAD-dependent DSBs [17, 20]. Bile acid mixtures modeling reflux produce similar ROS, apoptosis, and CAD-dependent *AF9* cleavage [18]. Ionizing radiation also triggers ROS, mitogen-activated protein kinase (MAPK) activation, caspase-3 cleavage, and DNA fragmentation, all suppressed by antioxidants [56].

#### Breakage at *MLL*

High cell density induces Southern blot evidence of *MLL* BCR cleavage, blocked by caspase-3 inhibition [57]. EBV LMP1 expression also promotes apoptosis and MAR/SAR-proximal *MLL* cleavage [57]. In SUNE1 cells, H₂O₂-induced site-specific DSBs in *MLL* were suppressed by ICAD overexpression, confirming CAD as the operative nuclease [17].

#### MAR/SAR-defined hotspots

Bioinformatic mapping identified MAR motifs adjacent to observed break clusters. In *AF9* (9p22), MAR-positive regions were enriched for apoptotic cleavage under oxidative stress, including that induced by bile acid exposure. Caspase inhibition abolished this enrichment [13, 19].

#### Misrepair leads to rearrangements

In *ABL1* (9q34), oxidative stress induced CAD-dependent intronic breaks at MAR/SARs, and de novo translocations were detected. Sequencing showed short microhomologies consistent with MMEJ [12].

#### Breakpoints coincide with leukemia patient BCRs

Oxidative stress-induced apoptosis in NPC models generated DNA breaks within *AF9* (9p22) and *ABL1* (9q34), both of which lie in regions frequently deleted in NPC [12, 18, 20]. Notably, these apoptosis-associated breaks occur within the breakpoint cluster region (BCR) of the *AF9* gene, with some breakpoints mapping to regions previously reported to translocate with *MLL* in acute lymphoblastic leukemia (ALL) [13, 20, 44]. Similarly, in the *ABL1* gene, mapped breaks were clustered within intron 1 in the BCRC, overlapping with patient-defined BCRs that give rise to the Philadelphia chromosome [12, 43].

### Synthesis

Collectively, NPC studies fulfill multiple mechanistic criteria:

**Temporal sequence:** apoptosis induction precedes break formation.

**Causality:** blocking caspase-3/CAD prevents the breaks.

**Positional specificity:** breaks occur at MAR/SARs overlapping tumor breakpoints.

**Pathological consequence:** misrepair via MMEJ yields structural variants.

Thus, apoptosis–MAR/SAR–MMEJ provides a coherent explanation for recurrent NPC rearrangements. Other processes such as replication stress [58, 59], ROS-induced base damage [60], aberrant activation-induced cytidine deaminase (AID) activity [61] may also contribute, but apoptosis-induced error-prone repair represents a convergent, experimentally validated pathway [12, 13, 17, 20, 56, 57]. In addition to DNA breakage and misrepair, inflammatory remodeling of the tumor microenvironment and associated immunosuppression may further facilitate the survival and expansion of apoptosis-surviving clones, linking mutational processes with selective clonal outgrowth [62].

## Broader implications in cancer

The apoptosis-induced rearrangement model has implications beyond NPC. The model proposes that a cell’s fate (survival or death) and genome structure interact to produce non-random mutations, reframing mutagenesis in several ways.

### A structural basis for recurrent fragile sites

**Fig. 4:**
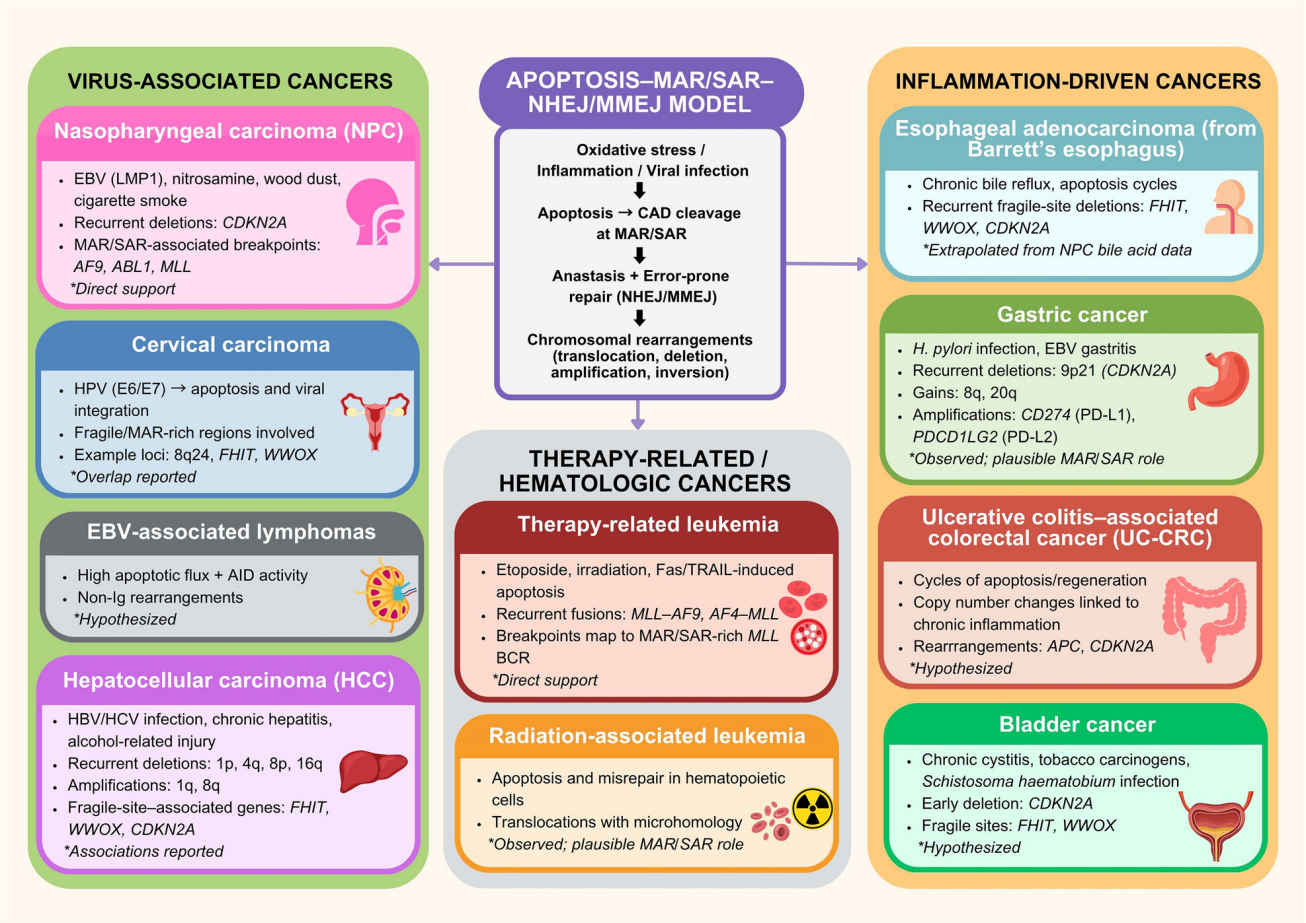
Cross-cancer schematic of apoptosis-induced chromosomal rearrangements via the MAR/SAR–NHEJ/MMEJ pathway. The central hub illustrates the apoptosis–MAR/SAR–NHEJ/MMEJ mechanism: oxidative stress, inflammation, or viral infection trigger caspase activation and CAD cleavage at MAR/SARs, followed by error-prone repair (predominantly MMEJ) that generates chromosomal rearrangements. Three cancer categories are shown: Virus-associated cancers: NPC (EBV; recurrent deletions of *CDKN2A*, breakpoints in *AF9, ABL1, MLL*), cervical carcinoma (HPV; fragile/MAR-SAR regions such as 8q24, *FHIT, WWOX*), EBV lymphomas (apoptosis + AID, non-Ig rearrangements), and hepatocellular carcinoma (HBV/HCV; recurrent deletions at 1p, 4q, 8p, 16q; amplifications at 1q, 8q). Inflammation-driven cancers: Barrett’s esophagus/adenocarcinoma (bile reflux; deletions in *CDKN2A, FHIT, WWOX*), gastric cancer (*H. pylori*/EBV; deletions at 9p21, gains at 8q and 20q, PD-L1/PD-L2 amplifications), ulcerative colitis–associated colorectal cancer (rearrangements in *APC, CDKN2A*), and bladder cancer (chronic cystitis, tobacco, *Schistosoma haematobium* infection; deletions in *CDKN2A, FHIT, WWOX*). Therapy-related/hematologic cancers: Leukemias with *MLL* fusions (etoposide-induced, microhomology junctions in *MLL, AF9*) and radiation-associated leukemias. Evidence categories (asterisks in figure) are indicated in the text as follows: *Direct support* indicates cases where MAR/SAR cleavage has been experimentally demonstrated in models, such as NPC and *MLL*-rearranged leukemia. *Overlap reported* indicates that fragile or MAR-rich loci coincide with viral integration sites or recurrent breakpoints, as seen in cervical cancer. *Hypothesized* indicates contexts where the mechanism is plausible but has not yet been experimentally mapped, for example ulcerative colitis–associated colorectal cancer (UC-CRC) or bladder cancer. *Extrapolated* indicates situations where evidence shown in NPC models has been extended to similar contexts, such as Barrett’s esophagus/adenocarcinoma with bile acid–induced apoptosis. *Observed* indicates cancers where rearrangements or microhomology patterns are documented but MAR/SAR involvement remains untested, including gastric cancer and radiation-associated leukemia. Created using Canva Pro.

Many cancers display recurrent breakpoints or fragile sites, regions frequently rearranged across patients. Classical fragile sites such as FRA3B in *FHIT* or FRA16D in *WWOX* have been linked to replication stress and DNA secondary structures [63–66]. NPC findings introduce MAR/SAR sequences as an alternative class of fragile sites driven by apoptosis [13, 19]. MAR/SARs are abundant throughout the genome, anchor chromatin loops, and often overlap with AT-rich scaffold-bound regions that are nuclease-sensitive [41, 42]. Figure 4 illustrates how apoptosis-induced breaks at MAR/SAR elements can undergo error-prone MMEJ repair, generating recurrent rearrangements. The model predicts that cancers with episodic apoptosis in precursor lesions may accumulate MAR/SAR breaks [13, 19].

In Barrett’s esophagus, esophageal adenocarcinoma, and colon cancer, chronic acid and bile exposure causes repeated epithelial apoptosis [28, 30]. These cancers show complex structural variants and focal deletions, some involving fragile-site genes such as *FHIT* and *WWOX* [67]. It is important to assess whether such breakpoints coincide with MAR/SAR motifs. In ulcerative colitis, a chronic inflammatory condition associated with an increased risk of colon cancer, regenerating epithelium undergoes repeated cycles of apoptosis [27]. Some colitis-associated copy number changes may reflect MAR/SAR-localized apoptotic breaks.

Virus-driven cancers also fit this framework. EBV in NPC and HPV in cervical cancer both induce apoptosis [31, 68]. In cervical carcinoma, HPV integration sites often lie within fragile regions [64, 69, 70], possibly enriched in MAR/SAR motifs vulnerable to HPV E6/E7–induced apoptosis and misrepair [31].

In hepatocellular carcinoma (HCC), chronic HBV or HCV infection drives apoptosis through oxidative stress, mitochondrial injury, and immune cytotoxicity [29]. Surviving hepatocytes may repair CAD-induced breaks [16] via error-prone pathways, creating recurrent deletions (1p, 4q, 8p, 16q) and amplifications (1q, 8q) [71]. Thus, HCC joins NPC and cervical carcinoma as virus-driven cancers that exemplify the apoptosis–MAR/SAR–MMEJ framework [12, 20].

These inflammation- and virus-driven contexts highlight a shared principle in which MAR/SARs act as structural determinants of breakpoints, providing a predictive lens for recurrent rearrangements across cancers.

### Apoptosis, anastasis, and cancer evolution

The model links anastasis, the recovery of cells from near-apoptosis, to genetic diversity [1]. Cancer evolution may occur not only through gradual mutation but also in bursts when dying cells survive with rearranged genomes [72]. This resembles chromothripsis, in which a chromosome shatters and reassembles in random order [73]. Initially attributed to mitotic accidents or micronuclei, chromothripsis may also result from apoptosis-survival [74]. If CAD cleaves DNA into fragments and the cell avoids death, fragments may rejoin randomly through MMEJ, producing chromothripsis [75]. Although direct evidence linking CAD-mediated apoptotic fragmentation to chromothripsis remains limited, this model provides a plausible mechanistic framework for catastrophic genome restructuring following incomplete apoptosis. Apoptosis-triggered rearrangements can thus be considered a structured form of chromothripsis, influenced by MAR/SARs [76].

Cells with intact p53 checkpoints rarely propagate such damage, since p53 induces arrest or death [75]. By contrast, cells with disabled p53 may survive with shattered genomes, enabling chromothripsis [75]. Many cancers, including NPC, exhibit compromised p53, as EBV proteins viral IL-10 and LMP1 impair p53 [77, 78]. This favors anastatic survival of apoptotic damage.

Immune suppression also contributes. Beyond generating DNA rearrangements, apoptosis-surviving cells must also evade immune-mediated elimination for structurally altered clones to persist and expand. An immunosuppressive niche enriched for T-bet⁺CD39⁺ Treg cells may increase tolerance of genomically altered cells. These Th1-like Treg cells, prevalent in many cancers including NPC, co-express *TBX21* (T-bet) and *ENTPD1* (CD39), thereby suppressing CD8⁺ T cells [62]. Mechanistic studies show that T-bet drives the intratumoral Th1-like Treg cell program, while CD39 mediates suppression [62]. Thus, apoptotic stress and immune escape may converge through CAD-mediated generation of structural variants and T-bet⁺CD39⁺ Treg cell-mediated suppression of immune clearance, allowing survival of rearrangement-bearing clones. Accordingly, the proposed model supports a punctuated equilibrium view of cancer evolution, in which bursts of mutations may arise from apoptotic escape rather than gradual accumulation.

### Extensions to other inflammation- and virus-associated cancers

Chronic inflammation is a well-known risk factor for multiple cancers, including colorectal, liver, pancreatic, and esophageal cancers, largely through ROS and cytokines that damage DNA and drive proliferation [79]. The apoptosis-associated mechanism proposed here further suggests that incomplete or aborted apoptosis can generate structural rearrangements through CAD-mediated DNA fragmentation and error-prone repair. This framework may apply broadly in cancers beyond NPC, including Barrett’s esophagus, HCC, gastric cancer, bladder cancer, and EBV-associated lymphomas.

#### Barrett’s esophagus and esophageal adenocarcinoma

In Barrett’s esophagus, bile acid-induced apoptosis is central to carcinogenesis [30]. Nasopharyngeal epithelial studies show that bile acid causes ROS and CAD-mediated breaks in *AF9* at 9p22 [18]. Repeated apoptotic injury may contribute to regional instability within chromosome 9p, and surviving Barrett’s cells may accumulate deletions such as 9p21 loss targeting *CDKN2A*, an early driver of esophageal adenocarcinoma [80].

#### Hepatitis and hepatocellular carcinoma (HCC)

In hepatitis or cholangitis, chronically inflamed hepatocytes or biliary cells undergo apoptosis; cells that regenerate might carry fused chromosomes or amplifications, as observed in cholangiocarcinoma [81]. In HCC, chronic HBV/HCV infection, alcohol-related hepatitis, and steatohepatitis produce repeated cycles of hepatocyte death and regeneration accompanied by oxidative stress and sublethal apoptosis [82]. This may provide fertile ground for CAD-mediated DNA cleavage at MAR/SAR regions [83], with error-prone repair potentially generating recurrent deletions (e.g., 1p, 4q, 8p, 16q) or amplifications (e.g., 1q, 8q) [84], many of which overlap with fragile-site-associated loci such as *FHIT*, *WWOX*, and *CDKN2A* [85, 86].

#### Chronic gastritis and gastric cancer

*Helicobacter pylori*-induced gastritis involves frequent epithelial apoptosis [32]. Some gastric cancers may arise from anastatic cells reassembling CAD-cleaved DNA. Researchers should look for MMEJ footprints and MAR/SAR proximity in gastric cancer structural variants. *H. pylori* gastritis and EBV-positive gastric cancer mirror NPC [87], with sustained oxidative stress, apoptosis, and recurrent genomic alterations [88]. Gastric tumors often show 9p21 deletions (*CDKN2A*), 8p loss, and gains of 8q and 20q [89]. EBV-positive gastric cancer also exhibits recurrent 9p24.1 amplifications involving *CD274* (PD-L1) and *PDCD1LG2* (PD-L2) [90]. Collectively, these features are consistent with the possibility that the apoptosis–MAR/SAR–MMEJ pathway may contribute to structural alterations in gastric cancer.

#### Bladder cancer

Bladder cancer develops in an inflamed environment caused by cystitis, tobacco carcinogens, or *Schistosoma haematobium* [91]. Early deletions of 9p21 (*CDKN2A*) and fragile loci such as *FHIT* and *WWOX* indicate recurrent breakage and misrepair [92]. Apoptotic turnover in urothelium under oxidative stress could create conditions permissive for CAD cleavage at MAR/SARs [93]. MMEJ repair may explain the structural losses observed during bladder tumorigenesis.

#### Lymphomas (EBV-associated subtypes)

EBV-associated Hodgkin lymphoma, diffuse large B-cell lymphoma, and NK/T-cell lymphoma may also fit this model [94]. Germinal centers already involve high apoptosis, amplified by EBV-driven cytotoxicity [95]. While AID explains canonical *Ig* translocations [96], some non-*Ig* rearrangements remain unexplained [97]. MAR/SAR cleavage may underlie these atypical lesions. Thus, the apoptosis–MAR/SAR–MMEJ pathway may complement AID in shaping the lymphoma genome.

### Hematological parallels (*MLL* fusions and others)

*MLL* rearrangements in leukemia are notable. *MLL* at 11q23 is frequently translocated in infant and therapy-related leukemias, often with *AF4*, *AF9*, *ENL*, and others [98]. A risk factor is exposure to topoisomerase II inhibitors such as etoposide [99], which induce apoptosis in hematopoietic cells. Etoposide-induced apoptosis produces DSBs at *MLL* [4], resembling NPC findings [17, 57]. Breakpoints cluster within an 8 kb BCR containing scaffold sites and AT-rich repeats [98], analogous to MAR/SAR-mediated NPC breaks. *MLL* junctions often show microhomology, implicating NHEJ/MMEJ [2]. Thus, the mechanism involving apoptotic DNA breakage followed by MMEJ may provide a unifying explanation for *MLL* translocations across different biological contexts. Targeting CAD [4] or Polθ-driven MMEJ may reduce such translocations [100, 101]. Another example is oncogenic translocations in bystander cells during radiotherapy, possibly arising from sublethal apoptosis and genomic reassembly [102, 103]. The implications span solid tumors and leukemias, where death and survival intersect, and where genomes may be reprogrammed in propagatable ways.

### Mutational signatures and structural variant prediction

Each mutational process leaves a genomic signature [104]. Apoptosis-induced rearrangements should show clustered variants with microhomology [105]. This pattern differs from rearrangements associated with HR deficiency or AID activity [105]. In sequencing data, one may detect multiple rearrangements mapping to MAR/SARs with microhomologies [12, 13, 19]. A pan-cancer study found many translocations with microhomology, implicating MMEJ [55]. Cross-referencing with MAR/SAR maps can identify apoptotic-origin events. In NPC, new rearrangements are likely to map to MAR/SARs [13, 19]. Thus, variant prediction could integrate MAR/SAR maps, flagging regions of high MAR/SAR density under oxidative stress. In silico analyses of selected genes within recurrent deletion hotspots in NPC identified predicted MAR/SAR elements associated with chromosomal breakage in NPC cell models [12, 13, 19], indicating that this strategy may be extended to guide the discovery of novel translocations and deletions.

### Therapeutic opportunities and preventive strategies

#### Targeting MMEJ/DNA polymerase θ (Polθ)

Cancers arising through apoptosis-survival may rely on alternative end-joining [106, 107]. Many overexpress Polθ, linked to poor outcomes [106, 107]. Polθ inhibitors are in development, especially for tumors lacking NHEJ or HR [107]. Tumor cells harboring rearrangements with MMEJ-like signatures may be particularly sensitive to Polθ inhibition [107].

#### Promoting complete apoptotic execution

Another vulnerability is the apoptotic pathway itself [35, 108]. Cells surviving apoptosis adapt by upregulating survival genes such as HSP70 or exportins [109]. In NPC, CAD breaks can be reduced by ICAD overexpression or caspase-3 inhibition [17, 20]. While sublethal apoptotic signaling promotes cell survival, partial apoptotic execution followed by error-prone end joining may fuel mutagenesis [34, 35]. Conversely, disabling ICAD may drive cells into complete apoptosis. However, direct ICAD targeting carries substantial risk.

Alternative pharmacological strategies exist to reinforce apoptosis. For example, phenethyl isothiocyanate plus paclitaxel synergistically enhanced α-tubulin acetylation, downregulated BCL2, upregulated BAX, and promoted PARP cleavage, amplifying death [110]. Such approaches may reduce the survival of cells carrying rearrangements, complementing Polθ inhibition or checkpoint targeting.

#### Exploiting checkpoint dependencies (ATR/CHK1)

Anastatic cells with complex karyotypes often rely on checkpoint kinases [111]. They may be especially dependent on ATR/CHK1, making them sensitive to ATR inhibitors [111]. Combining pro-apoptotic therapy with MMEJ inhibition could force cells into apoptosis, preventing mutagenic escape [107].

#### Preventive strategies

Prevention involves reducing chronic apoptosis-inducing stress. For example, proton pump inhibitors may mitigate GERD-induced Barrett’s apoptosis [112], and antiviral therapy may reduce EBV load in the nasopharynx [46].

Recognizing apoptosis-induced rearrangement as a mutational process identifies CAD and Polθ as key nodes [4, 107]. While globally inhibiting caspases or CAD is not viable, therapeutic windows may exist [4]. Transient caspase inhibition during radiation may protect normal cells while cancer cells lacking p53 still undergo apoptosis, although this approach remains experimental [4]. Alternatively, apoptosis sensitizers such as SMAC mimetics or BCL2 inhibitors could help ensure that tumor cells die completely [33]. These approaches highlight how understanding mutagenesis fate offers therapeutic opportunities [113].

**Fig. 5:**
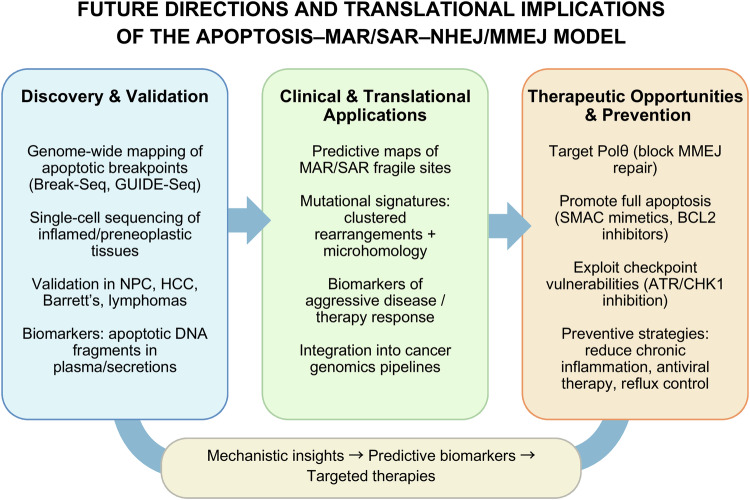
Translational implications and future directions of the apoptosis–MAR/SAR–NHEJ/MMEJ model. This conceptual roadmap highlights how the apoptosis-induced chromosomal rearrangement model can be extended from mechanistic discovery to clinical translation. Discovery & Validation (left): Genome-wide mapping of apoptotic breakpoints (e.g., Break-Seq, GUIDE-Seq) and MAR/SAR fragile sites, together with single-cell sequencing of inflamed or premalignant tissues to capture ongoing apoptotic DNA fragmentation. Validation in primary tumors (e.g., NPC, HCC, Barrett’s esophagus, lymphomas) and detection of apoptotic DNA fragments in plasma or secretions may establish “apoptotic footprints” as candidate biomarkers. Clinical & Translational Applications (center): Prediction of structural variant hotspots using MAR/SAR density maps and identification of mutational signatures characterized by clustered rearrangements with microhomology junctions. These features may serve as biomarkers of aggressive disease or therapy response, with integration into cancer genomics pipelines. Therapeutic Opportunities & Prevention (right): Targeting cancer dependencies created by apoptosis-induced rearrangements, including (i) inhibition of polymerase θ (Polθ) to block error-prone MMEJ, (ii) promoting full apoptotic execution using pro-apoptotic sensitizers (SMAC mimetics, BCL2 inhibitors) to prevent anastasis, and (iii) exploiting checkpoint vulnerabilities (ATR/CHK1 inhibition) in unstable post-apoptotic genomes. Preventive strategies include mitigating chronic inflammation, antiviral therapy, or reducing reflux-mediated bile acid exposure to lower sublethal apoptotic stress. Together, these avenues illustrate how the apoptosis–MAR/SAR–NHEJ/MMEJ framework integrates basic mechanistic biology with cancer genomics and clinical oncology, providing a roadmap for biomarker discovery and therapeutic innovation. Created using Canva Pro.

A summary of these approaches is in Table 1, while Fig. 5 provides a conceptual roadmap linking apoptosis-induced rearrangements to translation, biomarkers, and interventions.

**Table 1:** Therapeutic and preventive implications of apoptosis-associated misrepair.

Strategy	Target	Rationale	Potential application in cancer	Representative evidence/references
Enforce complete apoptosis	CAD–ICAD axis, apoptotic regulators (e.g., BCL2, SMAC mimetics)	Prevent survival of cells with fragmented DNA, reducing risk of misrepair	Enhance tumor cell clearance; limit chromosomal rearrangements	Genetic manipulation studies (e.g., CAD/ICAD, BCL2) or animal model studies; pharmacological enhancers of apoptosis [4, 33, 35, 110, 128]
Target error-prone repair	DNA polymerase θ (Polθ, MMEJ); ATR/CHK1 checkpoint kinases	Tumors surviving apoptosis rely on error-prone repair for DNA damage tolerance	Synthetic lethality in genomically unstable cancers; potential for combination with checkpoint blockade	Preclinical trials of Polθ inhibitors; ATR/CHK1 inhibition strategies [53, 107, 111]
Reduce apoptotic triggers	Inflammation (ROS, cytokines), chronic tissue damage, acid reflux	Limit cycles of apoptosis/regeneration and MAR/SAR breakage hotspots	Prevent accumulation of rearrangements in inflammation-driven cancers	Anti-inflammatory drugs, antioxidants, and reflux mitigation in experimental or clinical models [18–20, 56, 112, 135]
Viral prevention	HPV, HBV, HCV, EBV	Prevent virus-driven apoptotic DNA damage and integration-associated rearrangements	Reduce risk of cervical, liver, and virus-associated cancers	Public health vaccination programs; antiviral therapy outcomes [21, 29, 31, 46]
Biomarker applications	cfDNA structural variants, apoptotic DNA fragments; MAR/SAR-associated rearrangements	Misrepair signatures (e.g., clustered rearrangements, microhomology footprints) may serve as early cancer indicators	Early detection; prediction of structural variant hotspots; monitoring of therapy response and disease progression	MAR/SAR-associated rearrangements in NPC models [13, 19]; microhomology/ mutational signature analyses [12, 55, 104, 105]; cfDNA fragmentation as a biomarker [119, 120]

This table summarizes potential strategies to intercept apoptosis-induced chromosomal rearrangements, including approaches to enforce complete apoptosis, inhibit error-prone DNA repair, reduce apoptotic triggers, prevent virus-driven genomic damage, and apply biomarker analyses. Representative references include mechanistic, preclinical, clinical, or review evidence relevant to each strategy.

## Future directions

This apoptosis-centered model of chromosomal rearrangements, while strongly supported by current data, prompts several avenues for further research.

### Validating in patient tissues

Thus far, most direct evidence comes from cell models [12, 20]. Telomere shortening, demonstrated in NPC patient tissues [114], provides a genomic instability context that can promote apoptosis in nasopharyngeal epithelium. A key question is whether these tumors also harbor the MAR/SAR-linked breakpoints with microhomology that have been observed in NPC cell models [12, 13, 18–20]. While direct confirmation in patients is lacking, virus-driven genomic instability has been documented in other EBV-associated malignancies. For example, EBV LMP1 upregulates AID through Early Growth Response 1 (EGR1), leading to increased genomic lesions in B-cell lymphomas [115]. This highlights the principle that EBV can destabilize host genomes through distinct mechanisms. Importantly, structural variation analyses in NPC patients have already revealed recurrent gene fusions, including *YAP1*-*MAML2*, *PTPLB*-*RSRC1*, and *SP3*-*PTK2* [116]. What remains unclear is whether such rearrangements arise through the same MAR/SAR-linked apoptotic breakage and MMEJ repair mechanisms previously demonstrated in vitro at *AF9*, *ABL1*, and other MAR/SAR-rich loci [12, 13, 18–20].

Recent whole-genome sequencing of NPC tumors has mapped recurrent deletions and structural alterations [117]. Extending such analyses with algorithms to detect microhomology at junctions could directly test whether apoptosis-linked rearrangements occur in patients. Moreover, single-cell sequencing of nasopharyngeal epithelial cells from chronically inflamed tissues, made technically feasible by multiple annealing and looping-based amplification cycles (MALBAC) [118], may even capture cells in the act of acquiring such rearrangements.

Another angle is to look for apoptotic DNA fragments in plasma or secretions. Classical studies have shown that cells undergoing apoptosis release DNA ladders through CAD-mediated fragmentation [37]. Building on this, recent cell-free DNA (cfDNA) studies demonstrate that plasma DNA molecules carry characteristic fragmentation patterns that reveal their tissues of origin [119]. In patients with severe nasopharyngeal inflammation, one might even detect abnormal cfDNA fragments mapping to hotspot regions, representing a speculative ‘mutational biomarker’ of ongoing apoptotic damage, consistent with evidence that cfDNA fragmentation footprints can reflect cell type and chromatin structure [120].

### Genome-wide mapping of apoptotic breakpoints

Previous studies in NPC cell models have mainly examined candidate genes [12, 20]. A logical next step would be to perform unbiased, genome-wide mapping of apoptotic breakpoints in nasopharyngeal epithelial cells [121]. High-throughput methods originally developed for other purposes, such as Break-seq for mapping replication stress-induced DSBs or GUIDE-seq for profiling CRISPR off-target cleavage, could be adapted for this setting [121, 122]. Applying these approaches to apoptosis-induced DNA damage could generate a comprehensive landscape of apoptotic fragile sites, highlighting not only known NPC-associated loci but also novel genes and intergenic regions susceptible to CAD-mediated cleavage [123]. For instance, one might discover that other tumor suppressors implicated in NPC, such as *TP53*, *RB1*, or *PTEN*, harbor MAR/SAR-associated regions that are preferentially cleaved during apoptosis [44]. Importantly, such regions could subsequently be found deleted or rearranged in clinical NPC tumors, further supporting the mechanistic link between apoptotic cleavage sites and recurrent genomic alterations in cancer [124].

### Mechanistic nuances—role of other nucleases

CAD is the primary apoptotic nuclease [38], but other nucleases, such as endonuclease G (endoG) from mitochondria or DNase I-like enzymes, also act during cell death [125, 126]. These enzymes may contribute to the DNA breaks observed during apoptosis. Caspase inhibition assays indicated that caspase-3 (and thus CAD) is central [20], yet caspase-independent pathways, including AIF/endoG-mediated apoptosis, could also generate DSBs at comparable genomic locations [127]. Comparative studies of CAD-deficient cells (*DFFB* knockout) versus wild-type cells under stress would clarify whether residual breaks still occur at MAR/SAR regions in the absence of CAD [128]. The preferential cleavage of MAR/SAR sequences may be explained either by the physical localization of CAD at the nuclear matrix [39] or by intrinsic DNA features of MAR/SARs, such as bent DNA and AT-rich flexibility, that render them more accessible to CAD [129]. Detailed biochemical studies or ChIP-seq analysis of CAD during apoptosis could further elucidate these mechanisms [130].

### Interplay with other mutational processes

This apoptosis-mediated mechanism may interact with other known processes in NPC. For instance, EBV infection may contribute to aberrant AID activity in epithelial malignancies. In B cells, the EBV-encoded protein LMP1 has been shown to upregulate AID expression via EGR1, thereby promoting genomic instability [115]. Similar aberrant AID induction has also been documented in inflamed epithelial tissues under viral infection [131], supporting the plausibility of this mechanism in NPC. AID and CAD may potentially work in tandem, with AID creating nicks that lead to apoptosis, or CAD creating breaks that AID then erroneously fills (though this is less likely). Another contributing factor is telomere crisis, in which telomere shortening in some epithelial cancers leads to dicentric chromosomes and breakage-fusion-bridge cycles [132]. Telomere crisis itself induces p53-mediated apoptosis in many cells [133]; those that escape may carry both telomere fusions and CAD-induced internal breaks. Investigating NPC cells for signs of telomere dysfunction versus apoptotic breaks would help differentiate these sources of genomic chaos [134].

### Exploring other cancers

The model should be tested in other inflammation-linked cancers [135]. For example, bile acid–induced oxidative stress in Barrett’s epithelium, as shown by the induction of DNA damage under acidic bile exposure [136], may underlie breaks in *CDKN2A* (9p21) or *TP53* (17p), both of which are commonly affected in esophageal adenocarcinoma [137, 138]. Similarly, colitis-associated DNA lesions generated by reactive oxygen and nitrogen species, which accelerate tumorigenesis in colon tissue [25], may promote breaks in *APC* or other early colon cancer genes [139, 140]. Confirmation of such processes in these contexts would underscore a general principle applicable to many solid tumors [141].

In leukemia, the generation of chromosomal translocations such as *MLL–AF9* or *BCR–ABL1* can be modeled by DNA repair pathways that join DSBs with microhomology [51], raising the possibility that pro-apoptotic stimuli in hematopoietic stem cells in vivo could drive similar events. Elegant experimental systems could be devised, for example, by employing inducible caspase or CAD activity in specific cell populations, building on genome-wide translocation mapping approaches [142].

Chronic hepatitis–driven HCC also represents a strong candidate. Cycles of hepatocyte death and regeneration under HBV/HCV infection, alcohol-related injury, or steatohepatitis may create conditions favorable for CAD-mediated cleavage and error-prone repair [34]. Indeed, cytogenetic studies reveal recurrent deletions (1p, 4q, 8p, 16q) and amplifications (1q, 8q) in HCC [143], many of which overlap with fragile-site–associated genes such as *FHIT*, *WWOX*, and *CDKN2A* [85].

## Therapeutic opportunities and interventions

On the translational front, one could explore interventions in cell or animal models to interrupt this mutational process [107]. Polθ inhibitors, such as Novobiocin identified by Zhou et al. (2021), selectively kill HR-deficient tumor cells and may, by analogy, suppress the formation of translocations when NPC cells are subjected to repeated stress [144]. Conversely, overexpression of ICAD (which tightly binds and inhibits CAD) in nasopharyngeal epithelium could theoretically prevent chromosome breaks and thereby reduce malignant transformation in a chronic stress model [37]. Caution is warranted because blocking apoptosis can itself promote cancer by allowing damaged cells to survive [145]. The aim would be a nuanced approach to prevent partial apoptosis while still permitting either full cell death or complete survival without DNA damage [1]. One promising concept is the use of death-switch therapy, in which premalignant cells are equipped with a suicide gene that triggers complete cell death upon reaching a defined threshold of DNA damage, thereby avoiding the intermediate state of anastasis [146].

## Clinical correlations

The extent of apoptosis in preneoplastic lesions may correlate with genomic complexity in the ensuing tumor [147]. For NPC, persistent ear, nose, and throat (ENT) inflammation has been proposed as a biologically plausible contributor to nasopharyngeal carcinogenesis, although epidemiological evidence remains inconclusive [148], suggesting that biopsies of inflamed nasopharyngeal tissue with intense lymphocytic infiltration and potentially apoptotic debris would be valuable for histological investigation. If areas with greater apoptotic activity, for example as detected by TUNEL staining, correspond to foci of dysplasia or clonal genetic alterations, this would support the model at a histological level. While direct evidence in NPC is lacking, analogous findings in HCC show that DNA damage responses and genomic instability accumulate in preneoplastic lesions [149]. It would also be valuable to examine whether NPC patients have higher titers of nucleosomal DNA in circulation (a byproduct of CAD activity) than healthy controls, as elevated nucleosomes have been reported broadly in cancer patients [150].

In summary, an apoptosis-mediated route to mutagenesis has emerged as a compelling framework linking chronic inflammation, oxidative stress, and carcinogenesis. Foundational studies in NPC cell models [12, 13, 18–20] provided proof-of-principle that epithelial cells surviving sublethal apoptosis can acquire recurrent chromosomal aberrations. Yet much remains to be explored, including validation of this model in patient tissues, its broader applicability across tumor types, and the mechanistic contribution of chromatin architecture to breakpoint formation. In particular, the apparent susceptibility of MAR/SAR regions to caspase/CAD cleavage highlights the importance of nuclear matrix organization in shaping where apoptotic damage becomes fixed as heritable mutations. Looking ahead, translating this knowledge into strategies for cancer prevention and therapy will require integrating molecular dissection with clinical investigation [3]. Ultimately, establishing whether apoptotic DNA damage contributes to the mutational landscape of cancers such as NPC, HCC, and other inflammation-driven malignancies could open new avenues for biomarker development and therapeutic intervention.

## Conclusion

The convergence of mechanistic evidence from leukemia and experimental validation in NPC supports a refined understanding of cancer-associated genomic rearrangements. Rather than random consequences of “genomic instability,” chromosomal rearrangements in both hematologic malignancies and epithelial tumors may represent structured outcomes of incomplete apoptosis. In this model, DNA cleavage at MAR/SAR regions followed by error-prone rejoining generates structural mutations from a process normally intended to eliminate damaged cells.

This framework bridges molecular cell biology (nuclear matrix organization, caspase/CAD activity) with oncology (tumor evolution and genetic profiles). Supported by convergent findings in leukemia and NPC, it provides a plausible and potentially generalizable mechanistic explanation for recurring chromosomal breaks in both hematologic and epithelial malignancies and may extend to other inflammation-linked cancers in which cell death and survival are intertwined. Although NPC represents a particularly well-characterized solid-tumor context, parallel mechanistic features first defined in leukemia support the broader applicability of this model. Together, these findings suggest that mutations arise not solely by chance but within defined cellular and chromatin contexts, with the survival of partially apoptotic cells in both hematopoietic and epithelial settings representing a potential inflection point for malignant transformation. Although direct in vivo lineage-tracing evidence definitively linking apoptotic cleavage to clonal expansion remains limited, convergent mechanistic, cytogenetic, and genomic observations across leukemia and NPC support the biological plausibility of this framework.

From a translational perspective, therapeutic strategies that limit error-prone repair (for example, by targeting Polθ-mediated pathways) or reinforce complete apoptotic execution could reduce mutation-bearing survivor cells. Conversely, identifying the “apoptotic signature” of such mutations may yield biomarkers predictive of aggressive disease or therapy response.

In closing, this model underscores that genomic chaos is not entirely accidental but can arise from dysregulated cellular programs. NPC, positioned at the intersection of infection and chronic inflammation, exemplifies how apoptosis, normally tumor-suppressive, can paradoxically fuel oncogenic mutations when subverted. Appreciating the interplay between cellular context, chromatin structure, and mutational processes will be crucial as we continue to decode the cancer genome. More broadly, the apoptosis–MAR/SAR–MMEJ pathway may represent a common mechanistic theme shaping the mutational landscapes of multiple inflammation- and virus-associated cancers, offering new avenues for biomarker development and therapeutic intervention.

Taken together, these observations broaden our understanding of apoptosis beyond canonical execution and redefine how cell death programs interface with genome stability. Future work should delineate how apoptotic nucleases, anastasis, and error-prone end joining collectively shape cancer evolution, ideally through single-cell resolution and integrative chromatin-mapping approaches.
